# Association between newly initiated thiazide diuretics and hospitalization due to hyponatremia

**DOI:** 10.1007/s00228-020-03086-6

**Published:** 2021-01-15

**Authors:** Buster Mannheimer, Cecilia Fahlén Bergh, Henrik Falhammar, Jan Calissendorff, Jakob Skov, Jonatan D. Lindh

**Affiliations:** 1 Department of Clinical Science and Education at Södersjukhuset, Karolinska Institutet, Stockholm, Sweden; 2 Department of Internal Medicine, Section of Diabetes and Endocrinology, Södersjukhuset, Sjukhusbacken 8-10, 118 82 Stockholm, Sweden; 3 Department of Molecular Medicine and Surgery, Karolinska Institutet, Stockholm, Sweden; 4 Department of Endocrinology, Metabolism and Diabetes, Karolinska University Hospital, Stockholm, Sweden; 5 Department of Medicine, Karlstad Central Hospital, Karlstad, Sweden; 6 Department of Laboratory Medicine, Division of Clinical Pharmacology, Karolinska University Hospital Huddinge, Karolinska Institutet, Stockholm, Sweden

**Keywords:** Thiazides, Hospitalization, Hyponatremia, Adverse reaction

## Abstract

**Purpose:**

Thiazide diuretics are the most common origin of drug-induced hyponatremia. However, population-based studies on clinical outcomes are lacking. We therefore explored the time course and absolute risk of thiazide-associated hospitalization due to hyponatremia in Sweden.

**Methods:**

Population-based case-control study including patients hospitalized with a principal diagnosis of hyponatremia (*n* = 11,213) compared with controls (*n* = 44,801). Linkage of registers was used to acquire data. Multivariable regression was applied to explore time-dependent associations between thiazide diuretics and hospitalization due to hyponatremia. Attributable risks were calculated assessing the disease burden attributable to thiazides.

**Results:**

Individuals initiating thiazide treatment were exposed to an immediate increase in risk for hospitalization with adjusted odds ratio (aOR) (95% CI) of 48 (28–89). The associations gradually declined reaching an aOR of 2.9 (2.7–3.1) for individuals treated for longer than 13 weeks. The attributable risk of hyponatremia-associated hospitalization due to thiazides of any treatment length was 27% (3095/11,213). Among 806 patients initiating treatment < 90 days before hospitalization, hyponatremia could be attributed to thiazides in 754. Based on nationwide data, 616,678 individuals were initiated on thiazides during the 8-year study period suggesting an absolute risk of 0.12% (754/661,678) for subsequent hospitalization with a main diagnosis of hyponatremia.

**Conclusions:**

Thiazide diuretics attributed to more than one in four individuals hospitalized due to hyponatremia. The risk increase was very pronounced during the first month of treatment and then gradually declined, without returning to normal. However, the absolute risk for the development of hyponatremia demanding hospitalization may for most individuals be modest.

**Supplementary Information:**

The online version contains supplementary material available at 10.1007/s00228-020-03086-6.

## Introduction

Hyponatremia is the most common electrolyte imbalance affecting up to 30% of hospitalized patients [1]. Its clinical spectrum ranges from mild non-specific symptoms, such as nausea and fatigue, to severe symptoms such as confusion, seizures, and even death. One of the most common causes of hyponatremia is drug treatment [2]. Antidepressants, antiepileptic drugs, and several other drugs have been linked to an increased risk of severe hyponatremia [3–8]. Treatment with thiazide diuretics may be the most common origin of drug-induced hyponatremia [9]. Data suggest that hyponatremia due to thiazides often occurs shortly after initiation of therapy [10–13]. When initiating treatment, detailed knowledge on the time course and absolute risk are prerequisites for an optimal strategy and an adequate communication in order to avoid thiazide-induced hyponatremia. This information may also be important to evaluate the casual relation between thiazide treatment in a patient suffering from severe hyponatremia. The evidence on thiazide-induced hyponatremia is based on mostly small studies focusing on changes in serum sodium levels. However, population-based studies on clinical outcomes are lacking. We therefore explored the time course and absolute risk of thiazide-associated hospitalization due to hyponatremia in Sweden.

## Methods

This was a retrospective, population-based case-control study encompassing the adult Swedish population. Cases, defined as hospitalized subjects 18 years or older, with a first-ever primary diagnosis of hyponatremia (E87.1) or SIADH (E22.2) between October 1, 2006, and December 31, 2014, were identified in the National Patient Register (NPR). A first-ever diagnosis was defined as absence of a prior diagnosis (primary or secondary) of hyponatremia dating back to January 1, 1997. For each case, four controls without a previous diagnosis of hyponatremia were randomly identified using the Total Population Register. Controls were matched for age, sex, and municipality. Each case was assigned an index date based on the date of hospital admission. For controls, the index date was defined as the index date of their matched cases. During the study period (January 1, 1997, to December 31, 2014), all diagnoses in the NPR were coded according to the International Classification of Diseases, Tenth Revision (ICD-10). Concurrent and previous use of medications was identified using the Swedish Prescribed Drug Register (SPDR). The SPDR contains data on all prescribed and dispensed drugs in Sweden since July 1, 2005. Data on socioeconomic status was retrieved from the longitudinal integration database for health insurance and labor market studies register (LISA). The data collection process has been described in detail in previously [3]. The study was approved by the Regional Ethical Review Board in Stockholm. Due to its retrospective epidemiological nature, no informed consent was required.

### Variables

Thiazide dispensations were identified by Anatomical Therapeutic Chemical (ATC) codes starting with “C03A,” “C09BA,” “C09DA,” and “C03EA.” Drugs with ATC-codes “C08G,” “C07BB,” “C07C,” and “C09DX”, in many countries widely used, were not available on the Swedish market during the study period. Drug exposure was defined as a drug dispensation < 90 days prior to the index date. If the first thiazide dispensation in the 90 days period was preceded by a 1-year period without thiazide dispensations, the exposure was considered new, otherwise as ongoing. New exposures were further subdivided according to how many weeks (1–13 weeks) prior to the index date they had commenced. Confounding factors accounted for in the statistical analysis included concurrent medications, socioeconomic factors, and medical conditions identified using information from the NPR, the SPDR, and LISA. Selection of potential confounders was based on evidence with regard the potential to cause hyponatremia. Cardiovascular events were subdivided into those occurring within 90 days prior to the index date and older events. A complete list of exposure variables and confounding variables is provided in Supplemental table S1.

### Statistical analysis

The association between thiazide exposure and hyponatremia was investigated using univariable and multivariable logistic regression. In the primary analysis, the duration of exposure to thiazides was separated into newly initiated stratified by weeks of exposure (1–13 weeks) or ongoing, as described above. In secondary analyses, odds ratios (ORs) were calculated for thiazide exposure regardless of duration and separated into new (0–90 days) vs. ongoing.

To quantitate the disease burden and absolute risk of thiazide-associated hyponatremia, adjusted ORs were used to calculate attributable risk percentages. The attributable risk percentage was defined as the percentage of cases in a population that is attributable to the exposure of interest. This statistic indicates the percentage of cases that could theoretically be prevented by removing the exposure from the population. To estimate the overall number of individuals started on thiazides during the corresponding time period, nationwide data from the Swedish Prescribed Drug Register was used [14]. *p* values < 0.05 were considered statistically significant. All calculations were performed using R version 3.6.1.24.

## Results

During the 8-year study period, we found 11,213 individuals, over 18 years of age, with a principle diagnosis of hyponatremia. In addition, 44,801 matched controls were included.

The majority were of female gender (72%) and the median age (range) was 76 (18–103) years. Table 1 shows a selection of medical characteristics and current medications, including thiazide diuretics, at index date among the study population. Overall, cases had more comorbidities and used more medications than controls. The most common accompanying medical conditions were previous malignant disease, ischemic heart disease, and diabetes.

**Table 1:** Medical characteristics in addition to thiazide use among cases and controls at index date

	Number of total cases (*n* = 11, 213)	Number of total controls (*n* = 44, 801)	*p* value
Diagnosis			
Malignancy	3096 (27.6%)	9149 (20.4%)	< 0.001
Ischemic heart disease	2186 (19.5%)	6290 (14.0%)	< 0.001
Diabetes mellitus	1939 (17.3%)	5277 (11.8%)	< 0.001
Alcoholism	1764 (15.7%)	833 (1.9%)	< 0.001
Congestive heart failure	1453 (13.0%)	3541 (7.9%)	< 0.001
Cerebrovascular disease	1448 (12.9%)	3533 (7.9%)	< 0.001
COPD	1125 (10.0%)	1576 (3.5%)	< 0.001
Renal disease	489 (4.4%)	888 (2.0%)	< 0.001
Liver disease	421 (3.8%)	332 (0.7%)	< 0.001
Medications			
Antidepressants	2817 (25.1%)	5745 (12.8%)	< 0.001
Antiepileptic drugs	1061 (9.4%)	1128 (2.5%)	< 0.001
Furosemide	1735 (15.5%)	5487 (12.2%)	< 0.001
Calcium channel blockers	2283 (20.4%)	6432 (14.4%)	< 0.001
Betablockers	4175 (37.2%)	11,363 (25.4%)	< 0.001
Proxy for frailty			
Number of dispensed drugs 90 days prior to index date			
< 4 drugs	2215 (20.0%)	22,892 (51.1%)	< 0.001
4–7 drugs	3421 (30.5%)	12,967 (28.9%)	0.0013
8–12 drugs	3558 (31.7%)	7010 (15.6%)	< 0.001
> 12 drugs	2019 (18.0%)	1932 (4.3%)	< 0.001
Previous hospitalization ≥ 3 days duration	4852 (43.3%)	9477 (21.2%)	< 0.001
Thiazide diuretics			
Total	4364 (38.9%)	6103 (13.7%)	< 0.001
Newly initiated*	806 (7.2%)	246 (0.5%)	< 0.001
Ongoing use	3558 (31.7%)	5857 (13.1%)	< 0.001

*COPD* chronic obstructive pulmonary disease

*During the 90 days before index date

**Fig. 1:**
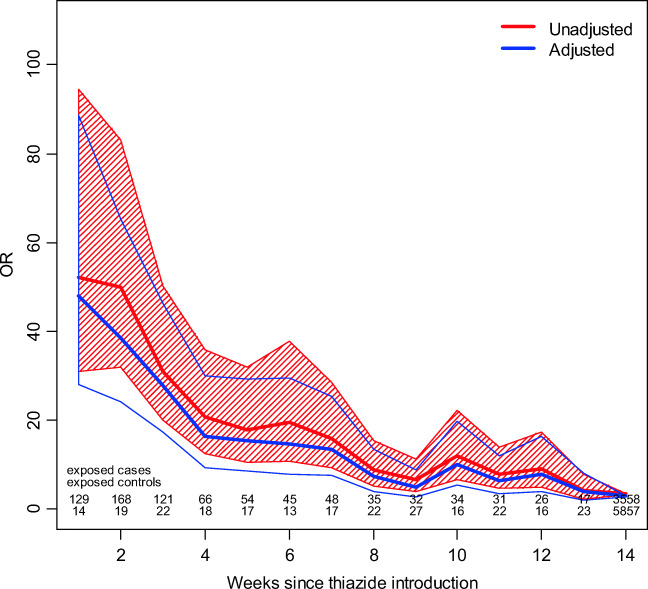
Unadjusted and adjusted odds ratios (ORs), accompanied with a 95% confidence intervals for hospitalization due to hyponatremia week by week

The crude OR (95% CI) for exposure to thiazides regardless of treatment duration was 4.0 (3.9–4.2). After adjustment for potential confounders, the OR decreased slightly (3.4 (3.2–3.7)). To further investigate the temporal association between initiation of thiazide diuretics and hospitalization due to hyponatremia, we investigated the associations week by week (Fig. 1). In general, crude ORs were similar to adjusted ORs (aORs). Individuals initiating thiazide treatment were exposed to an immediately increased risk for hospitalization at week 1 reaching an aOR (95% CI) of 48 (28–89). The associations then gradually declined reaching an aOR of 4.1 (2.0–81) by week 13. The aOR for individuals treated for longer than 13 weeks was 2.9 (2.7–3.1).

To estimate the corresponding disease burden, we analyzed the percentage of cases that were hospitalized due to hyponatremia potentially attributed to thiazide treatment (attributable risk percentage). For individuals with any thiazide exposure regardless of treatment duration (*n* = 4364), the attributable risk (95% CI) was 27.6 (26.8–28.4%) indicating that 3095 individuals may be hospitalized due to a hyponatremia attributed to thiazides over the 8-year study period. In addition, we calculated the disease burden among the minority of patients exposed for newly initiated treatment, i.e., thiazides initiated < 90 days from hospitalization (*n* = 806). Among 806 patients with newly initiated treatment, hyponatremia could be attributed to thiazides in 754. To analyze the absolute risk of being hospitalized following newly initiated treatment, we related the disease burden to the number of individuals initiated on thiazides during the corresponding 8-year study period. According to nationwide data, 616,678 individuals were initiated on thiazides suggesting an absolute risk increase of 0.12% (754/616,678) for hospitalization with a main diagnosis of hyponatremia.

## Discussion

This is the first large-scale study investigating the clinical implications of thiazide-induced hyponatremia. It revealed that thiazide diuretics may be the precipitating cause in more than one in four cases hospitalized due to hyponatremia. The risk was almost 50-fold increased during the first week of treatment and then gradually declined. Patients with ongoing treatment were exposed to more than a three-fold increase in risk.

Even though thiazide-induced hyponatremia is well described, population-based evidence on the epidemiology remains scarce. One well conducted population-based cohort study found that the hazard ratios for developing moderate (> 125– < 130 mM) and severe (≤ 125 mM) hyponatremia after initiation of thiazide diuretics were both above 8. However, neither time-dependent effects nor clinical outcomes were presented [15]. There is some evidence of an increased risk associated with newly initiated treatment. In a controlled experimental study on rechallenge of patients with confirmed thiazide-induced hyponatremia, Friedman et al. [11] demonstrated a significant decrease in serum sodium levels within 24 h of administering a single dose of thiazide diuretics. Furthermore, in a case series including 129 individuals with severe thiazide-induced hyponatremia, Sonnenblick et al. [16] reported that in most cases, the hyponatremia developed within 14 days of initiation of treatment, and Leung et al. [13] demonstrated a 60% increase in relative risk of hyponatremia (serum sodium < 130 mmol/L at any point over a period of 10 years) in 220 patients initiating thiazide diuretics compared to patients started on other antihypertensive drugs. The risk peaked during the first 3 months of treatment but continued to be elevated during the entire 10-year study period. The risk for hospitalization was not significantly elevated.

The current population-based approach including the entire population of Sweden is the first large study investigating important clinical consequences of thiazide-induced hyponatremia. The population-based approach permitted us to investigate the fraction of hospitalizations that was attributed by thiazide diuretics. We found that more than one in four (3104/11,213) hospitalizations with a main diagnosis of hyponatremia may be caused by thiazides. This underscores the important role of thiazides among the group drug-induced hyponatremia contributing with a substantial disease burden.

Figure 1 illustrates a very pronounced association between individuals exposed for a thiazide initiated < 90 days and hospitalization due to hyponatremia in the first weeks of thiazide treatment compared to individuals with longer duration of treatment. The pattern suggests a strong causal relationship. The subsequent analysis of the attributable risk among 806 patients initiating treatment < 90 days before hospitalization in fact indicated thiazides to be the precipitating cause in 94% (754/806). However, is the association strong enough to further support routine monitoring of sodium levels 1–2 weeks after thiazide initiation which has been suggested [17]? To answer this question, we assessed the absolute risk for hospitalization with a main diagnosis of hyponatremia by relating to the overall number of individuals started on thiazides during the corresponding time period. Based on nationwide data from the Swedish Prescribed Drug Register [14], 616,678 individuals were initiated on thiazides during the 8-year study period suggesting a low absolute risk of 0.12%. However, it is important to note that patients hospitalized with a main diagnosis of hyponatremia most likely represent a modest proportion of all patients with severe hyponatremia. In a Danish study reporting on the validity of ICD codes for hyponatremia, the sensitivity for a main or secondary diagnosis of hyponatremia was approximately 30% in patient with serum sodium below 115 nmol/L [18]. Nevertheless, the absolute risk of severe hyponatremia secondary to thiazide treatment appears to be low. The finding of an almost 50-fold increased risk of hospitalization during the first week of treatment highlights that electrolyte monitoring of individuals with newly initiated thiazides should be performed on wide indications. Furthermore, the results emphasize the need for serum electrolyte controls also in patients developing symptoms consistent with hyponatremia long after initiation of treatment. This is especially important in risk groups, i.e., elderly females [12].

Interestingly, the larger group of individuals with thiazide treatment > 90 days (*n* = 3558) was associated with an increased risk as well. Although more moderate (OR 2.9), the prevailing risk emphasizes the need to consider thiazide withdrawal also in most patients with severe hyponatremia associated with ongoing treatment unless other underlying causes are identified. Evidence suggests that this may not always be the case. Thus, Fahlén Bergh et al. [19] showed that thiazides were not withdrawn in 25% of patients despite being hospitalized due to hyponatremia.

The main finding of this study was the time-dependent, vastly increased risk associated with newly initiated thiazide treatment. These results are in line with previous evidence [11, 15, 20], suggesting a direct rather than cumulative effect of thiazides in precipitating hyponatremia. Data suggesting a dose-independent effect may indicate an idiosyncratic effect [21]. On the other hand, risk factors have been identified that seem to predispose some individuals for hyponatraemia. Known risk factors include old age, female sex, and low body weight. In addition, a genetic variant has been identified affecting the function of the apical prostaglandin transporter of the distal nephron [22]. Similar temporal patterns have been shown with regard to several antihypertensives [8] as well as other drug classes [4, 5, 7]. SSRIs and venlafaxine constitute one important example where the risk may be exclusively related to newly started treatment [3, 23, 24]. However, for thiazides, the risk of severe hyponatremia appears to persist with ongoing treatment. This could signal a cumulative effect of thiazides in some individuals, but a more reasonable explanation is perhaps that modest hyponatremia develops upon initiation of treatment, but that additional factors introduced at a later stage cause a further drop in serum sodium or a worsening of symptoms, resulting in hospitalization.

A major strength of the present study is the population-based design and the inclusion of all individuals (*n* > 4000) with thiazide-associated hospitalizations due to hyponatremia in Swedish population as a whole. The main limitation is the lack of available plasma sodium concentrations. However, since we only considered patients with a main diagnosis of hyponatremia, we made sure that only patients with clinically relevant hyponatremia were included. Clinically, the principal reason for hospitalization may be more adequate as compared to studies depending on hyponatremia as a secondary diagnosis, diagnoses made outside the hospital setting in the secondary or primary care [25], or inclusion based on low sodium values regardless of associated symptoms [24]. To further motivate our study design, a validation of the principal diagnosis of hyponatremia was assessed. We found that 89% had been hospitalized mainly due to symptoms of hyponatremia. The mean plasma sodium concentration was 121 mmol/L, i.e., profound hyponatremia, further emphasizing the clinical relevance of the study design [3]. A wide range of comorbidities and drugs may influence the risk for hyponatremia. While adjusting for a wide range of variables that may be related to hyponatremia, we cannot exclude the possibility of residual confounding from factors not accounted for. For example, we lacked information on body weight which would have been useful as low body weight is a known risk factor for hyponatremia. Furthermore, initiation of treatment, in this case thiazides, is always associated with a condition that motivated such treatment, and part of the temporal pattern in hyponatremia risk may relate to a worsening of underlying disease, rather than the treatment itself. Another limitation is the case-control study design which in several regards is less robust as compared to the cohort design, especially so when aiming to draw conclusions with regard to absolute risks. Although combining estimated attributable risk with population statistics on drug exposure allowed us to draw some conclusions regarding the absolute risk of being hospitalized due to hyponatremia after starting thiazide treatment, a cohort study design would have been more robust for several reasons. Most importantly, a cohort design directly analyses the incidence in the entire population under study while the current approach relies on an indirect estimation based on the comparison of thiazide exposure between cases and a limited fraction of controls. In addition, a cohort design would have enabled estimation of absolute risk estimates for all risk factors included in the model, not only the thiazides now addressed by means of attributable fractions. Such information could potentially have been used for individualized risk prediction in patients eligible for thiazide therapy. Finally, the present study was based on dispensed drugs, but dispensation dates do not necessarily translate to exposure dates, as first drug intake may take place at a later point in time, or not at all.

In conclusion, thiazide diuretics attributed to more than one in four individuals hospitalized due to hyponatremia. The risk was very pronounced during the first month of treatment and then gradually declined, without returning to normal. However, the absolute risk for the development of hyponatremia demanding hospitalization may for most individuals be modest.

## Supplementary information

Table S1 A complete list of variables included in the multivariate logistic regression model. (DOCX 17 kb).

## Data Availability

Data will be made available due to reasonable requests.
