# Supplementary material for: Association between newly initiated thiazide diuretics and hospitalization due to hyponatremia
Source: Eur J Clin Pharmacol. 2021 Jan 15;77(7):1049–55. doi: 10.1007/s00228-020-03086-6 (PMC8184529; doi:10.1007/s00228-020-03086-6)
Supplement: Supplementary file 1 — A complete list of variables included in the multivariate logistic regression model. (DOCX 17 kb). [file 228_2020_3086_MOESM1_ESM.docx]

**Supplemental table S1:** Variables included in logistic regression model

| **Variables** | **Codes** |
| --- | --- |
|  | **ATC codes beginning with** |
| **Drugs of primary interest** |  |
| Thiazide diuretics | C03A, C09BA, C09DA, C03EA, C08G, C07BB, C07C, C09DX |
| **Antiepileptic drugs** |  |
| Carbamazepine | N03AF01 |
| Oxcarbazepine | N03AF02 |
| Phenytoin | N03AB02 |
| Valproate | N03AG01 |
| Lamotrigine | N03AX09 |
| Levetiracetam | N03AX14 |
| Gabapentin | N03AX12 |
| **Other antihypertensive drugs** |  |
| Furosemide | C03CA01 |
| Torasemide | C03CA04 |
| Bumetanide | C03CA02 |
| Eplerenone | C03DA04 |
| Spironolactone | C03DA01 |
| Amiloride | C03DB01, C03EA |
| Agents affecting the renin-angiotensin system | C09 |
| Calcium channel blockers | C08, C07FB02, C09DB |
| **Antibiotics** |  |
| Fluoroquinolones | J01MA |
| Macrolides | J01FA |
| Trimethoprim sulfamethoxazole | J01EE |
| **Antidepressants** |  |
| SSRIs | N06AB |
| Tricyclic antidepressants | N06AA |
| Other antidepressants | N06AX |
| **Other drugs** |  |
| Amiodarone | C01BD01 |
| Desmopressin | H01BA02 |
| Proton pump inhibitors | A02BC, A02BD06 |
| Antipsychotics (excluding lithium) | N05A excluding N05AN |
| NSAIDs | M01AA, M01AB, M01AC, M01AE, M01AG, M01AH, M01AX01, N02AJ08, N02AJ19 |
| Statins | C10AA, C10BA02, C10BA03, C10BA05, C10BA06 |
|  | **ICD10 codes beginning with** |
| **Renal diseases** |  |
| Renal insufficiency | N17-19, procedure codes DR016, DR024, KAS00, KAS10, KAS20 |
| **Infections** |  |
| Sepsis | A41 |
| Pneumonia | J18 |
| Meningitis | G00-G07 |
| **Heart and vascular diseases** |  |
| Ischemic heart disease | I20-25 |
| New ischemic heart disease event* | I20-24 |
| Congestive heart failure | I50 |
| Cerebrovascular diseases | I60-64, I69 |
| New cerebrovascular event* | I60-64 |
| **Gastrointestinal diseases** |  |
| Pancreatic disease | K85, K860-1 |
| Inflammatory bowel disease | K50-51 |
| Liver diseases | K70-77 Procedure codes JJB, JJC |
| **Other diseases** |  |
| Hypothyroidism | E03, E06.3 |
| Malnutrition | E43.9, E41.9 |
| COPD | J44 |
| Pulmonary embolism | I26 |
| Malignancy | C |
|  | **Combination of ATC- and ICD-10 codes, each beginning with** |
| Alcoholism | **ATC:** N07BB03, N07BB04, N07BB01, N07BB05, N07BB  **ICD10:** E244, F10, G312, G621, G721, I426, K292, K70, K860, O354, P043, Q860, T51, Y90-91, Z502, Z714 |
| Adrenal insufficiency | **ATC:** H02AA, H01BA  **ICD10**: E27.1, E27.2, E27.3, E27.4, E25 |
| Diabetes mellitus | **ATC:** A10  **ICD10:** E10-E14 |
| **Socioeconomic factors** |  |
| Education | Increasing levels of education from 1-6, continuous variable |
| Income | Income in Swedish crowns during 1 year, continuous variable |
| Unemployment | Number of days, continuous variable |
| **Proxy for frailty** |  |
| Drug use | Number of dispensed drugs 90 days prior to index date, categorised into <4, 4-7, 8-12 and >12 drugs |
| Duration of hospitalization | ≥3 days |

*During the 90 days before index date

SSRIs, selective serotonin reuptake inhibitors. NSAIDs, non-steroidal anti-inflammatory drugs. COPD, chronic obstructive pulmonary disease.
